# A hybrid deep learning model for sentiment analysis of COVID-19 tweets with class balancing

**DOI:** 10.1038/s41598-025-97778-7

**Published:** 2025-07-30

**Authors:** Md. Alamin Talukder, Md. Ashraf Uddin, Suman Roy, Partho Ghose, Smita Sarker, Ansam Khraisat, Mohsin Kazi, Md Momtazur Rahman, Musawer Hakimi

**Affiliations:** 1 Department of Computer Science and Engineering, International University of Business Agriculture and Technology, Dhaka, Bangladesh; 2 School of Information Technology, Deakin University, Burwood Campus, Melbourne, Australia; 3 Department of Computer Science and Engineering, Jagannath University, Dhaka, 1100 Bangladesh; 4 Department of Statistics, Hajee Mohammad Danesh Science and Technology University, Dinajpur, 5200 Bangladesh; 5 Department of Pharmaceutics, College of Pharmacy, King Saud University, P.O. Box-2457, 11451 Riyadh, Saudi Arabia; 6 Department of English and Modern Languages, International University of Business Agriculture and Technology, Dhaka, Bangladesh; 7 Department of Computer Science, Samangan University, Northeast Aybak, Samangan Province Afghanistan; 8 School of Information Technology, Crown Institute of Higher Education, Canberra, Australia

**Keywords:** Sentiment analysis, Deep learning, BERT, LSTM, Tweets, COVID-19, Computer science, Information technology

## Abstract

The widespread dissemination of misinformation and the diverse public sentiment observed during the COVID-19 pandemic highlight the necessity for accurate sentiment analysis of social media discourse. This study proposes a hybrid deep learning (DL) model that integrates Bidirectional Encoder Representations from Transformers (BERT) for contextual feature extraction with Long Short-Term Memory (LSTM) networks for sequential learning to classify COVID-19-related sentiments. To enhance data quality, advanced text preprocessing techniques, including Unicode normalization, contraction expansion, and emoji conversion, are applied. Additionally, to mitigate class imbalance, Random OverSampling (ROS) is employed, leading to significant improvements in model performance. Before applying ROS, the model exhibited lower accuracy and inconsistent performance across sentiment categories. After balancing the dataset, accuracy for binary classification increased to 92.10%, with corresponding precision, sensitivity, and specificity of 92.10%, 92.10%, and 91.50%, respectively. For three-class sentiment classification, accuracy improved to 89.47%, with precision, sensitivity, and specificity of 89.80%, 89.47%, and 94.10%, respectively. In five-class sentiment classification, accuracy reached 81.78%, with precision, sensitivity, and specificity of 82.19%, 81.78%, and 95.28%, respectively. These findings demonstrate the efficacy of combining deep learning-based sentiment analysis with advanced text preprocessing and class balancing techniques for accurately classifying public sentiment related to COVID-19 across multiple sentiment categories.

## Introduction

The swift growth of social media has significantly changed how people interact, communicate, and obtain information^1^. Platforms such as Twitter, Facebook, and Instagram have become integral components of daily life, offering users a space to express opinions, share experiences, and engage in discussions on diverse topics, including health-related issues^2^. The COVID-19 pandemic further amplified the reliance on social media, as individuals sought real-time updates, disseminated information, and expressed their concerns regarding the crisis^3^. During lockdowns and quarantine measures, social media served as a critical medium for staying connected, receiving news, and discussing public health policies^4^. While social media provides an avenue for information sharing and public discourse, it also presents significant challenges, particularly in the spread of misinformation and disinformation^5^. The proliferation of misleading or false narratives, especially concerning public health crises like COVID-19, has had far-reaching psychological and societal implications^6^. Studies indicate that exposure to inaccurate information on social platforms can contribute to heightened anxiety, public distrust, and panic. Inaccurate statistics and unverified claims circulating on digital platforms have further complicated efforts to manage the pandemic effectively^7^. To mitigate the adverse effects of misinformation, it is essential to develop automated mechanisms capable of detecting and filtering unreliable content before it spreads widely.

Sentiment analysis has emerged as a powerful tool for examining public opinion and behavioral trends on social media^8^. By analyzing user-generated content, researchers can assess public sentiment, detect emotional trends, and gauge societal attitudes toward key events^9^. Sentiment analysis has diverse applications, ranging from opinion mining in market research to assessing public reactions to government policies^10^. Given the dynamic nature of social media, real-time sentiment analysis presents valuable insights for policymakers, healthcare professionals, and researchers. In the context of COVID-19, sentiment analysis can play a crucial role in understanding public perception, detecting misinformation, and assessing the emotional impact of health crises^11^. Various machine learning (ML) and deep learning (DL) techniques have been proposed to enhance sentiment classification and misinformation detection. Traditional ML approaches, such as decision tree (DT), random forest (RF), and multinomial naive bayes (MNB), have demonstrated effectiveness in text classification tasks but often struggle with complex, context-dependent language patterns. Deep learning models, particularly transformer-based architectures such as BERT (Bidirectional Encoder Representations from Transformers), have significantly improved sentiment analysis performance by capturing contextual semantics and linguistic nuances.

Despite advancements in sentiment analysis, several challenges remain. Many studies predominantly focus on optimizing model accuracy without adequately addressing issues such as dataset preparation and class imbalance. Furthermore, sentiment analysis of COVID-19-related discussions often relies on binary classification (positive vs. negative), overlooking the nuanced nature of public sentiment, which may also include neutral or mixed emotions. Additionally, most existing studies are based on relatively small datasets, limiting the generalizability of their findings. Addressing these limitations is essential for developing robust and scalable sentiment analysis frameworks capable of capturing the complexity of social media discourse.

This study aims to bridge these research gaps by employing an advanced sentiment analysis framework that integrates ML and DL models. Specifically, we leverage the BERT-LSTM model, a hybrid approach combining BERT’s contextual understanding with LSTM’s sequential processing capabilities, to enhance sentiment classification performance. Our research introduces an improved preprocessing pipeline to refine data quality and implements data balancing techniques to mitigate class imbalance issues. By benchmarking the proposed model against various ML and DL approaches, we provide a comprehensive evaluation of sentiment analysis effectiveness in real-world scenarios. Additionally, this study explores the broader implications of sentiment analysis in crisis management, emphasizing its role in combating misinformation and improving public health communication during global emergencies.

### Objectives and motivations

The main goal of this study is to improve sentiment analysis of COVID-19-related tweets by addressing key gaps in existing literature, particularly in preprocessing techniques, data balancing, model performance evaluation, and real-world applications. Traditional sentiment analysis approaches often struggle with the noisy and context-dependent nature of social media data, leading to suboptimal classification performance. While previous studies have explored ML and DL models for sentiment classification, they frequently overlook the issue of class imbalance, which might result in less generalizability and biased model predictions. Many sentiment datasets exhibit skewed distributions, where certain sentiment classes (e.g., neutral or positive) dominate, leading to poor performance in minority classes. Additionally, a comprehensive evaluation of different models across multiple sentiment classes remains limited.

Motivated by these challenges, this study introduces an advanced preprocessing pipeline designed to improve data quality, along with effective data balancing methods to mitigate class imbalance and enhance model fairness. Furthermore, we systematically benchmark the hybrid model against various ML and DL approaches to establish its effectiveness in handling complex sentiment nuances. Beyond methodological advancements, this research aims to provide actionable insights for improving public health communication by using sentiment analysis to identify misinformation and assess public sentiment during health crises. By bridging these gaps, our study contributes to inventing more reliable, unbiased, and context-aware sentiment analysis models, ultimately aiding in informed decision-making and crisis management.

### Research questions

To overcome the difficulties in sentiment analysis of tweets pertaining to COVID-19, this study explores the following key research questions:

- What are the most effective preprocessing techniques for analyzing COVID-19-related tweets to ensure high-quality sentiment analysis?
- How does the performance of our hybrid (BERT-LSTM) model compare to other traditional ML models and DL models in the sentiment classification of COVID-19 tweets?
- How can the findings from this study be applied to improve public health communication and manage misinformation on social media?

## Research contributions

This study makes several significant contributions toward addressing the research questions:

- **Robust preprocessing framework:** We develop an advanced text preprocessing pipeline incorporating tokenization, stopword removal, lemmatization, and noise reduction, enhancing data quality for improved sentiment classification in social media discussions.
- **Effective data balancing strategy:** To address class imbalance, we apply Random Over-Sampling (ROS), improving model performance and generalizability across different sentiment categories.
- **Hybrid deep learning architecture:** We propose a hybrid model that integrates BERT’s contextual feature extraction with LSTM’s sequential learning, leading to enhanced sentiment classification accuracy.
- **Comprehensive model evaluation:** We conduct a systematic comparison of traditional ML and DL models, utilizing TF-IDF for ML models and BERT embeddings for DL models, across binary, three-class, and five-class sentiment classification tasks.
- **Implications for public health:** The findings contribute to the development of automated sentiment analysis tools for misinformation detection, assisting public health organizations in crisis communication and response strategies.

The structure of the paper is as follows: A review of relevant literature on the sentiment analysis of COVID-19 tweets is provided in Sect. “Related works”. The suggested technique and study materials are described in full in Sect. “Methodology”. The performance analysis and outcomes are covered in Sect. “Results”. A comparison of the suggested model with current methods is given in Sect. “Discussion”, along with an emphasis on its implications, drawbacks, and possible future paths. The study is finally concluded in Sect. “Conclusion”.

## Related works

The efficacy of deep learning models in sentiment analysis has been shown in recent research, especially when it comes to examining public debate on social media during the COVID-19 pandemic. Researchers have leveraged deep learning techniques to classify sentiments expressed in COVID-19-related tweets, providing insights into public perception and behavioral trends Various studies have explored sentiment classification across diverse demographic groups using real-time social media data, highlighting the role of deep learning in understanding opinion dynamics^12^. Additionally, a Few-Shot Transfer Learning (FSTL-SA) framework has been proposed for sentiment polarity detection, showcasing its potential in low-resource scenarios where labeled data is limited^13^. These advancements underscore the significance of deep learning in extracting meaningful sentiment insights from large-scale social media data.

A research by Bacsarslan et al.^14^ used sentiment analysis on Twitter tweets to examine public opinion about the epidemic. The study used both ensemble learning techniques (Majority Voting, Probabilistic Voting, and Stacking) and individual machine learning models (Decision Tree, K-Nearest Neighbor, Logistic Regression, Naïve Bayes, and Random Forest). Tweets were transformed using Word2Vec, Doc2Vec, TF-IDF, and Bag of Words. The results indicated that ensemble models outperformed individual classifiers, with Stacking and Doc2Vec achieving the highest accuracy (89%), demonstrating the effectiveness of ensemble techniques.

A stacking ensemble classifier was established in a study by Jayapermana et al.^15^ to improve the accuracy of categorizing COVID-19 vaccine-related comments on Twitter. Support Vector Machine (SVM), Random Forest, and Logistic Regression were all used as first-level learners in this approach, with Logistic Regression acting as the meta-learner. According to evaluation results, the suggested model obtained an F1-score of 85%, 86% accuracy, 85% precision, and 86% recall. The innovative aspect of the study was its ability to enhance classification performance by combining many machine learning models using a stacking technique, which proved successful in multi-class classification problems.

Dobler et al.^16^ conducted an initial study analyzing English Twitter posts about the COVID-19 pandemic to examine opinion differences based on cultural backgrounds. The research aimed to classify tweets into collectivist and individualistic cultures. TF-IDF was used for feature extraction, while a pre-trained BERT model served as the neural network for classification. Sentiment analysis categorized posts as positive, negative, or neutral. The study also explored potential educational implications and adaptive measures for different cultural groups. The model achieved 73.5% accuracy, 71.6% recall, 72.2% precision, 71.0% F1-score, 72.2% sensitivity, and 74.6% specificity, demonstrating its effectiveness.

Bacsarslan et al.^17^ conducted sentiment analysis on two datasets consisting of tweets related to the coronavirus. The datasets were processed using two vectorization methods: Term Frequency-Inverse Document Frequency (TF-IDF) and Word2Vec. They trained several models for sentiment classification, which included individual machine learning models (Decision Tree, K-Nearest Neighbor, Naïve Bayes, and Support Vector Machine), deep learning models (Long Short-Term Memory and Recurrent Neural Network), as well as ensemble learning methods (Stacking and Majority Voting). The primary evaluation metric used was accuracy. Ultimately, the Stacking ensemble model, which combined LSTM and RNN, achieved the highest accuracy of 86.4% on the COVID-19 dataset.

In order to analyze and evaluate the sentiment of tweets shared during the COVID-19 pandemic, Tareq et al.^18^ used a Bidirectional Long Short-Term Memory (BiLSTM) model within a Recurrent Neural Network (RNN) to predict sentiment classes: Negative, Positive, and Neutral. Positive and negative tweets differed slightly, according to the results, suggesting that more awareness is required to lessen negativity. The BLSTM model attained an accuracy of 86.15% and was able to predict sentiment classes. The findings demonstrated that BLSTM could accurately predict and adjust to the emotion of textual input.

To enhance sarcasm detection, Balaji et al.^19^ created a unique framework called SARCOVID, which used ensemble approaches and hierarchical transfer learning. SARCOVID outperformed conventional techniques with lower bias and higher accuracy when tested on a COVID-19 dataset. The study found that sarcasm was prevalent in online conversations regarding COVID-19, underscoring the need for sophisticated sarcasm detection methods. SARCOVID outperformed current models during testing, achieving an accuracy of 0.61 on the Sarcasm Corpus V2. In addition to improving sentiment analysis for lively online discussions, our approach offered insightful information about the complex sentiment expressions found on many social media sites.

Many studies have been conducted on the sentiment classification of text data, particularly in relation to COVID-19. Researchers are actively seeking improved solutions for analyzing public sentiment during the pandemic. Upon reviewing the existing literature, we notice that some authors utilized ML models exclusively, while others incorporated both DL models to gain deeper insights into public sentiment and assist government efforts in managing public dissatisfaction. However, most of these studies focused on binary class categories, with only a few considering three-class categories. In addition, the datasets used for sentiment analysis were generally small in scale. In our research, our primary focus is on employing DL techniques for classifying multi-class sentiments concerning COVID-19.

## Methodology

**Fig. 1:**
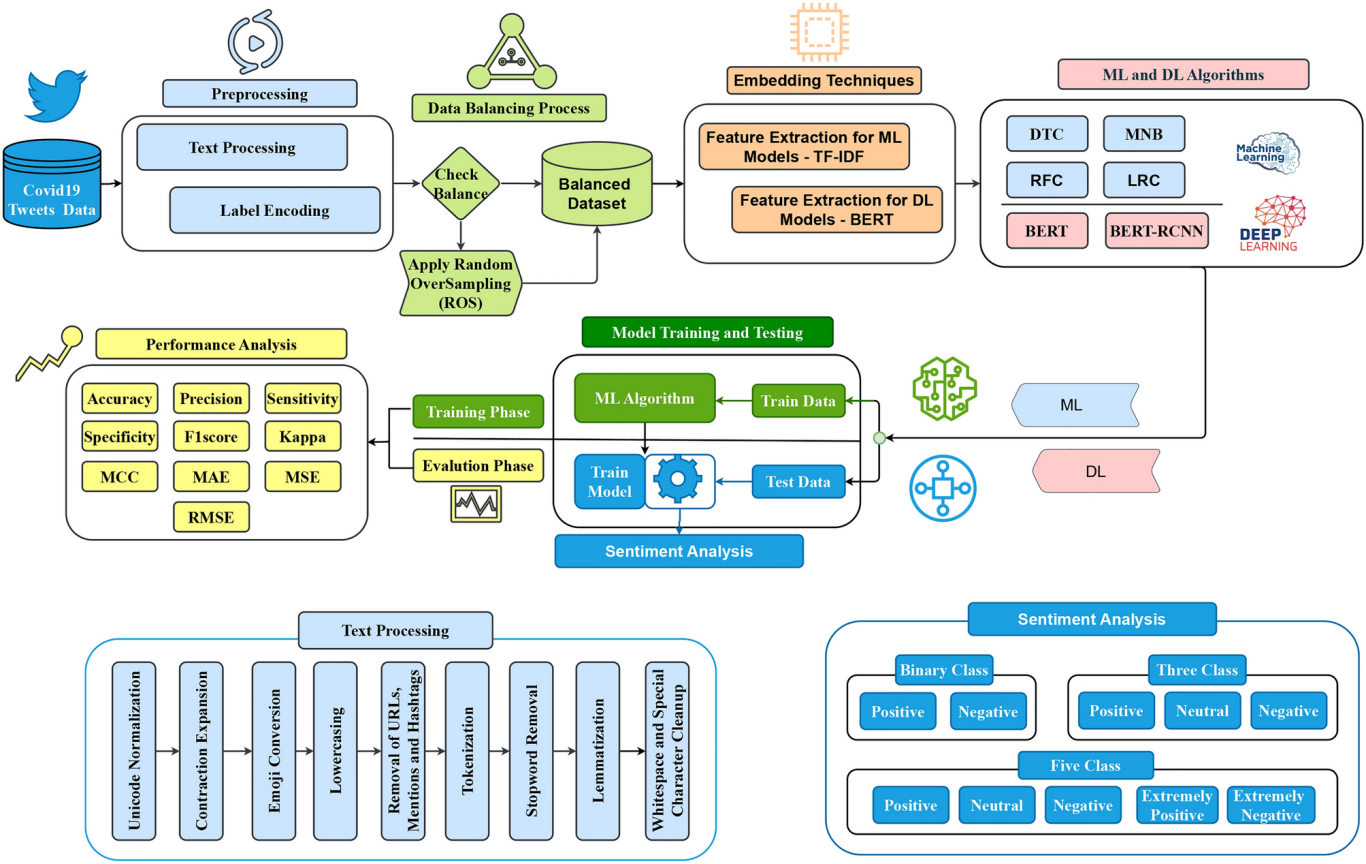
The proposed hybrid (BERT-LSTM) architecture for sentiment analysis.

The proposed methodology for sentiment analysis of COVID-19 tweets follows a structured pipeline encompassing preprocessing, data balancing, feature extraction, model training, and performance evaluation, as illustrated in Fig. 1. Initially, raw tweets undergo comprehensive text preprocessing, including Unicode normalization, contraction expansion, emoji conversion, lowercasing, removal of URLs, mentions, and hashtags, tokenization, stopword removal (excluding negations), lemmatization, and whitespace normalization. Label encoding is then applied to transform sentiment labels into numerical values. To address class imbalance, the dataset undergoes a balancing process using Random OverSampling (ROS) to ensure an equal representation of sentiment classes. DL models employ BERT embeddings for feature extraction, whereas ML models use TF-IDF. The dataset is divided into subgroups for testing and training. ML models (DTC, RFC, MNB, and LRC) are trained using features that have been retrieved. In order to train DL models (BERT, BERT-LSTM), contextual embeddings are used. The following metrics are used in performance evaluation: ACC, PREC, REC, F1, SPEC, KAPPA, MAE, MSE, and RMSE. Finally, the models classify sentiments into binary (positive, negative), three-class (positive, neutral, negative), and five-class (positive, neutral, negative, extremely positive, extremely negative) categories, providing a comprehensive sentiment analysis framework for assessing public opinion on COVID-19-related discussions across social networks.

### Dataset description

During the COVID-19 lockdown, public sentiment varied significantly, influencing social discourse across different regions. To capture these variations, we utilized a dataset comprising 44,955 tweets, compiled from Twitter messages in several different nations. The dataset was sourced from Kaggle, a publicly accessible and open-source repository. These tweets reflect public emotions, opinions, and concerns during the pandemic, making the dataset valuable for sentiment analysis research. The dataset utilized in this study comprises four key attributes: Location, TweetAt, OriginalTweet, and Sentiment. These attributes provide essential contextual information, enabling an in-depth analysis of the attitude of tweets gathered throughout the COVID-19 outbreak. The Location attribute specifies where the tweet was posted, while TweetAt records the timestamp. The OriginalTweet contains the actual text analyzed, and Sentiment categorizes the tweet into Positive, Neutral, or Negative, forming the basis for classification tasks in this research.

#### Sentiment analysis task

This dataset was employed in a sentiment analysis experiment, where we aimed to classify tweets into different sentiment categories. Sentiment analysis is crucial for understanding public opinion, tracking emotional trends, and assessing responses to events like the COVID-19 pandemic. The dataset was utilized in three different classification tasks:

- Binary sentiment: Distinguishing between Positive and Negative sentiments.
- Three-class sentiment: Identifying Positive, Neutral, and Negative Sentiments.
- Five-class sentiment: Expanding classification to Extremely Positive, Positive, Neutral, Negative, and Extremely Negative categories for more granular sentiment detection.

A thorough analysis of the dataset distribution across various sentiment classification tasks is given in Table 1, which also highlights the differences in the quantity of examples available for testing and training.

**Table 1:** Dataset distribution for sentiment analysis.

Sentiment classes	Training set	Test set
Binary classification (Negative, Positive)		
Negative	23,111	2252
Positive	18,046	1546
Three-class classification (Negative, Neutral, Positive)		
Negative	15,398	1633
Neutral	7713	619
Positive	18,046	1546
Five-class classification (Negative, Neutral, Positive, Extremely Negative, Extremely Positive)		
Negative	9917	1041
Neutral	7713	619
Positive	11,422	947
Extremely negative	5481	592
Extremely positive	6624	599

#### Challenges in dataset

The challenges in the sentiment dataset include class imbalance, which drives it problematic for the model to properly comprehend underrepresented classes. Additionally, the dataset may contain noisy or mislabeled data, affecting the quality of the training process. Variations in the data, such as different formats or resolutions, can also introduce inconsistencies that hinder model performance. Addressing these challenges often requires thorough text preprocessing, data balancing, and careful evaluation to ensure reliable and accurate results.

### Text preprocessing

In this sentiment analysis classification experiment, a structured text preprocessing pipeline is applied to refine raw tweets for ML models. The steps include Unicode normalization to remove accents and special characters, contraction expansion, and emoji conversion to retain sentiment value. Text is standardized to lowercase, and social media artifacts such as URLs, user mentions, and hashtags are removed. Tokenization, stopword removal (excluding negations), and lemmatization are performed to refine linguistic representation. Finally, redundant spaces and special characters are eliminated to ensure clean formatting. This preprocessing enhances the quality of textual data, enabling effective sentiment classification.

1. **Unicode normalization:** Ensures consistency by removing accents and special characters.
2. **Contraction expansion:** Converts contractions (e.g., “can’t” to “cannot”) into their full forms.
3. **Emoji conversion:** Replaces emojis with descriptive text to preserve sentiment meaning.
4. **Lowercasing:** Standardizes text by converting all characters to lowercase.
5. **Removal of URLs, Mentions, and Hashtags:** Eliminates social media artifacts irrelevant to sentiment analysis.
6. **Tokenization:** This process divides the text into individual words or tokens for further analysis.
7. **Stopword removal:** Removes common stopwords while retaining negations critical for sentiment analysis.
8. **Lemmatization:** Reduces words to their base forms to improve generalization.
9. **Whitespace and special character cleanup:** Eliminates redundant spaces and non-essential symbols for clean formatting.

### Label encoding

In order to translate categorical sentiment labels into numerical values that machine learning models can comprehend effectively, label encoding is used in this experiment. The following encoding schemes are used based on the sentiment granularity:

1. **Binary classification**: The sentiment labels are encoded (Table 2)as follows:

**Table 2:** Binary label encoding.

Sentiment category	Encoded value
Negative	0
Positive	1
2. **Three-class classification**: For a three-class sentiment analysis, the labels are encoded (Table 3) as:

**Table 3:** Three-class label encoding.

Sentiment category	Encoded value
Neutral	0
Positive	1
Negative	2
3. **Five-class classification**: For a more fine-grained classification, five distinct sentiment categories are encoded (Table 4) as:

**Table 4:** Five-class label encoding

Sentiment category	Encoded value
Neutral	0
Positive	1
Extremely negative	2
Negative	3
Extremely positive	4

This encoding process ensures that the sentiment categories are effectively represented as numerical values, making them suitable for ML model training and evaluation.

### Data balancing

Data balancing refers to the technique used to address class imbalances in a dataset, which can direct to biased learning models. When the class distribution is highly skewed, the model may become biased toward the majority class, resulting in poor performance, especially for the minority class. To mitigate this issue, we applied Random Oversampling (ROS), a technique where the minority class samples are randomly duplicated to correspond to the number of samples in the majority class.

Mathematically, ROS involves the following steps:

Given a dataset with classes $C_1, C_2, \dots , C_k$ and corresponding class counts $N(C_1), N(C_2), \dots , N(C_k)$, let $N_{\text {majority}} = \max (N(C_1), N(C_2), \dots , N(C_k))$ be the count of the majority class. ROS creates new instances by randomly selecting samples from the minority class, with the substitute, until the number of samples in each class equals $N_{\text {majority}}$.

The motivation for using ROS in sentiment analysis is that it allows the model to better learn from underrepresented classes, which is essential for tasks such as sentiment classification where certain sentiments (e.g., “Neutral” or “Extremely Positive”) may appear less frequently. By balancing the classes, the model can generalize better, improving its performance across all sentiment categories.

After applying ROS, the dataset distribution for the training data (Table 5) is as follows:

**Table 5:** Resampled class distribution after data balancing (Training dataset).

Sentiment class	Original class distribution	Resampled class distribution
Negative	23111	23111
Positive	18046	23111
Neutral	7713	18046
Positive	18046	18046
Negative	15398	18046
Extremely negative	5481	11422
Negative	7713	11422
Positive	11422	11422
Extremely positive	6624	11422

### Dataset partition

Our dataset is divided into two primary sections: the testing and training datasets. There are 3,798 samples in the test dataset and 41,157 samples in the training dataset. In order to generate a validation set, we retained 90% of the training dataset for training and used 10% for validation. The dataset distribution that was produced is displayed in Table 6.

**Table 6:** Dataset distribution in the experiment set.

Dataset	Number of samples
Training dataset	37041
Validation dataset	4116
Test dataset	3798

### Embedding techniques

In our experiment, we employ two distinct embedding techniques to represent text data effectively, each suited for different types of models: BERT embeddings for deep learning models and TF-IDF embeddings for ML models. BERT embeddings are pre-trained, contextualized word embeddings generated by the BERT model. BERT processes text bidirectionally, capturing the nuanced meanings of words based on their surrounding context. This technique enables the model to understand complex linguistic structures and context-dependent word representations, making it particularly effective for tasks like sentiment analysis, where understanding the context is critical. BERT embeddings are utilized in our deep learning models, such as the BERT-LSTM, to enhance performance by leveraging rich, context-aware representations. In contrast, TF-IDF embeddings are used with ML models to capture the importance of words within a document relative to the entire dataset. TF-IDF focuses on identifying terms that are unique to specific documents, providing a sparse representation that is particularly useful for feature-based models like RFC and LRC. These embeddings are applied in our ML models to improve performance in tasks where feature extraction is key. Thus, BERT embeddings are used for deep learning models, while TF-IDF embeddings are applied for ML models, maximizing each strategy’s performance according to its advantages.

The distinct embedding techniques used in the experiment illustrate how MLand DL models leverage various types of feature representations as follows:

#### Feature extraction for DL models

In this study, we utilize the BERT tokenizer as part of the feature extraction process for building the BERT-LSTM model. For a variety of NLP applications, BERT, a pre-trained transformer model, has demonstrated exceptional efficacy. For feature extraction, we employ the BERT tokenizer to convert input sentences into tokenized representations that BERT can process. Specifically, we use the BertTokenizer from the transformers library, initializing it with the model identifier “bert-base-uncased”. This tokenizer converts text into a sequence of tokens, which are then mapped to their respective token IDs. These token IDs are used as inputs to the BERT model, which generates contextualized embeddings for each token in a sentence.

The tokenizer processes the sentences with truncation and padding to ensure a consistent sequence length, specified as 128 tokens. Padding is added to sentences shorter than this length, and longer sentences are truncated. The tokenizer outputs several components, including input_ids, attention_mask, and token_type_ids. The input_ids represent the tokenized words, while the attention_mask indicates which tokens are padding (0) and which are actual data (1). The token_type_ids are used to differentiate between different sentences in tasks like question-answering. These components are converted into tensors and moved to the appropriate device (GPU or CPU) for model processing.

We also define a collate_fn function, which prepares the batch for training by handling tokenization and padding for each sentence, converting the corresponding labels to tensor format, and ensuring that the output is compatible with the BERT model. A PyTorch DataLoader is used to load the dataset efficiently, with batching and shuffling enabled to improve training performance.

The main benefit of using BERT for feature extraction in the BERT-LSTM model is its ability to generate rich, context-dependent word embeddings. BERT captures the nuances of language by processing sentences bidirectionally, meaning that each word’s meaning is influenced by both the words preceding and following it. This feature enables the model to comprehend intricate linguistic patterns, enhancing performance in jobs where context comprehension is essential, such as sentiment analysis. The BERT-LSTM model improves the model’s capacity to identify both local and global dependencies in the text by combining BERT embeddings with a CNN architecture to further extract hierarchical features from the tokenized input. This hybrid method is very successful for sentiment classification tasks, particularly in complex datasets with a wide range of language expressions, because it combines the pre-trained knowledge of BERT with the spatial feature extraction power of CNNs.

#### Feature extraction for ML msodels

Feature extraction plays a crucial role in ML by converting raw data into a structured format suitable for ML algorithms. In NLP tasks, such as sentiment analysis, one of the most commonly used techniques for extracting features from text data is **TF-IDF**. This method transforms textual information into numerical vectors by assessing the significance of words within a document in relation to the entire dataset.

TF-IDF is computed in two key steps:

1. **Term frequency (TF):** This measures how often a specific word appears in a document. The higher the frequency, the more relevant the word may be to the document’s context. It is mathematically expressed as:
  $$\begin{aligned} \text {TF}(w, d) = \frac{\text {Number of times word } w \text { appears in document } d}{\text {Total words in document } d} \end{aligned}$$
  where $w$ represents a word, and $d$ denotes a document.
2. **Inverse document frequency (IDF):** This evaluates the uniqueness of a word across multiple documents. Words that appear frequently across many documents contribute less to distinguishing content, while rarer words carry more weight. IDF is defined as:
  $$\begin{aligned} \text {IDF}(w) = \log \left( \frac{N}{\text {df}(w)} \right) \end{aligned}$$
  where $N$ is the total number of documents, and $\text {df}(w)$ represents the number of documents containing the word $w$.

The final **TF-IDF score** is computed by multiplying these two values:

$$\begin{aligned} \text {TF-IDF}(w, d) = \text {TF}(w, d) \times \text {IDF}(w) \end{aligned}$$

This score highlights the relevance of a word in a specific document relative to the entire dataset.

For our implementation, we utilize the TfidfVectorizer from the sklearn.feature_extraction.text module to transform textual data into numerical feature vectors. The TfidfVectorizer automatically calculates TF-IDF scores for words across the dataset and generates a sparse matrix, where each row corresponds to a document, and each column represents a unique term in the vocabulary. This matrix is then used as input for ML models, such as LRC or DTC.

The primary benefits of using TF-IDF for feature extraction in ML models include:

- **Weighting term importance**: By emphasizing rare and informative terms while down-weighting common words, TF-IDF helps ML models focus on the most relevant features.
- **Handling high-dimensional data**: TF-IDF can handle the high-dimensional nature of text data efficiently by transforming the text into numerical vectors that preserve semantic meaning, even with sparse representations.
- **Improved accuracy for text classification**: TF-IDF allows the models to understand and utilize the contextual importance of words within the corpus, leading to better performance in classification tasks, such as sentiment analysis.

By using TF-IDF for feature extraction, we can complement our deep learning models with traditional ML models, allowing for a hybrid approach that benefits from both data-driven and expert-engineered feature sets.

### Machine and deep learning algorithms

In our experiments, we used ML and DL models to identify the best-performing approach. Performance of BERT-LSTM was compared with other DL models to assess its effectiveness. Traditional ML techniques, including DTC, MNB, RFC, and LRC, were evaluated. DL models tested include BERT and BERT-LSTM, offering a comprehensive comparison between ML and DL approaches.

#### Decision tree classifier (DTC)

The Decision Tree classifier is a rule-based model that recursively partitions the dataset by selecting features that best differentiate the target classes. The selection process aims to reduce impurity at each node, which is typically measured using entropy or the Gini index. The mathematical formulations for these impurity measures are:

$$\begin{aligned} H(D) = -\sum _{i=1}^{k} p_i \log _2 p_i \end{aligned}$$

where $p_i$ represents the probability of class $i$ in dataset $D$.

$$\begin{aligned} Gini(D) = 1 - \sum _{i=1}^{k} p_i^2 \end{aligned}$$

where $p_i$ is the probability of class $i$ in $D$.

The feature with the minimum entropy or Gini index is selected at each step to create splits in the dataset. Decision Trees are widely used due to their interpretability, ability to handle various data types, and capacity to capture non-linear relationships.

#### Multinomial naive bayes (MNB)

The Multinomial Naive Bayes classifier applies Bayes’ theorem to estimate the probability of a class given a set of input features. It assumes that feature occurrences are independent given the class. The probability of class $C$ given an input $X$ is calculated as follows:

$$\begin{aligned} P(C \mid X) = \frac{P(C) \prod _{i=1}^{n} P(x_i \mid C)}{P(X)} \end{aligned}$$

where:

- $C$ is a class label.
- $X = (x_1, x_2, \dots , x_n)$ represents the input feature vector.
- $P(C \mid X)$ is the posterior probability of class $C$.
- $P(C)$ is the prior probability of class $C$.
- $P(x_i \mid C)$ is the likelihood of feature $x_i$ occurring in class $C$.
- $P(X)$ is the normalizing constant.

Naive Bayes is computationally efficient, performs well with high-dimensional data, and is particularly effective when dealing with word frequency-based features.

#### Random forest classifier (RFC)

Random Forest is an ensemble learning method that constructs multiple Decision Trees and aggregates their predictions to enhance accuracy. The algorithm employs bootstrap aggregation (bagging), where each tree is trained on a random subset of the dataset. The final prediction is determined by majority voting. The mathematical representation of Random Forest is:

$$\begin{aligned} {\hat{y}} = \frac{1}{T} \sum _{t=1}^{T} f_t(X) \end{aligned}$$

where: - $T$ represents the total number of trees. - $f_t(X)$ is the prediction from the $t$-th tree given input $X$.

Random Forest reduces overfitting compared to a single Decision Tree and provides feature importance scores, which help in understanding the impact of individual variables.

#### Logistic regression classifier (LRC)

Logistic Regression is a statistical model used for binary classification tasks. It predicts the probability that an input belongs to a particular class using the logistic function:

$$\begin{aligned} P(y=1 \mid X) = \frac{1}{1 + e^{-w^T X + b}} \end{aligned}$$

where:

- $P(y=1 \mid X)$ is the probability that the input $X$ belongs to class 1.
- $w$ is the weight vector.
- $X$ is the feature vector.
- $b$ is the bias term.
- $e$ is the Euler’s number (natural logarithm base).

The model parameters ($w$ and $b$) are optimized by minimizing the log-likelihood loss function using gradient descent. Logistic Regression is favored for its simplicity, interpretability, and effectiveness in scenarios where the relationship between features and output is approximately linear.

#### BERT model

BERT (Bidirectional Encoder Representations from Transformers) is a deep learning model based on the Transformer architecture. It leverages self-attention mechanisms to process text in a bidirectional manner, considering both preceding and succeeding words in a sentence. The self-attention mechanism is computed as:

$$\begin{aligned} \text {Attention}(Q, K, V) = \text {softmax}\left( \frac{Q K^T}{\sqrt{d_k}} \right) V \end{aligned}$$

where: - $Q$ (query), $K$ (key), and $V$ (value) are matrices derived from input embeddings. - $d_k$ represents the dimension of the key vectors.

BERT undergoes pre-training through two key tasks: Masked Language Modeling (MLM), where certain words in a sentence are masked and predicted, and Next Sentence Prediction (NSP), where the model learns sentence relationships. Fine-tuning BERT on downstream tasks such as sentiment analysis enhances its ability to capture contextual meanings in text data.

By utilizing bidirectional context, BERT significantly improves performance across various NLP tasks, outperforming traditional word embedding models in terms of language understanding.

#### BERT-LSTM model

The BERT-LSTM model is a hybrid deep learning approach that combines the Bidirectional Encoder Representations from Transformers (BERT) with a Long Short-Term Memory (LSTM) network to enhance sentiment analysis of COVID-19-related tweets. BERT is pre-trained on large-scale textual data and generates contextual embeddings that capture the semantic meaning of words within a sentence. However, while BERT effectively models word-level dependencies, it does not explicitly retain sequential information across longer contexts. To address this, we integrate an LSTM layer after BERT, which allows the model to capture long-term dependencies and improve sentiment classification accuracy. The bidirectional LSTM (BiLSTM) further enhances the learning process by processing text sequences in both forward and backward directions, ensuring a richer understanding of sentiment patterns in tweets.

This hybrid architecture improves upon traditional machine learning models and standalone deep learning models like BERT by leveraging both contextual embeddings and sequential modeling. Unlike conventional machine learning approaches, which require manual feature extraction, BERT-LSTM automatically learns meaningful representations from raw text. Additionally, compared to BERT-only models, the inclusion of LSTM ensures that sequential dependencies are preserved, making it particularly effective for short, informal texts like tweets. By employing this model, we achieve superior performance in sentiment classification, capturing nuanced emotions and opinions expressed on social media regarding the COVID-19 pandemic.

#### Mathematical explanation of BERT-LSTM model

The BERT-LSTM model combines BERT’s powerful contextual word representations with LSTM’s ability to model long-range dependencies in sequential data. Mathematically, the model can be expressed as follows:

#### BERT embedding representation

Given an input tweet represented as a sequence of tokens:

1 $$\begin{aligned} X = [x_1, x_2,..., x_n] \end{aligned}$$

BERT encodes each token $x_i$ into a contextualized embedding $h_i$ using a multi-layer Transformer-based architecture:

2 $$\begin{aligned} H = BERT(X) = [h_1, h_2,..., h_n] \end{aligned}$$

where $H \in {\mathbb {R}}^{n \times d}$, and $d$ is the hidden dimension (typically 768 for BERT-base). The special [CLS] token embedding, denoted as $h_{CLS}$, is often used as a sentence representation.

#### LSTM for sequential modeling

The embeddings from BERT are fed into a bidirectional LSTM (BiLSTM) layer, which processes the sequence in both forward and backward directions:

3 $$\begin{aligned} & \overrightarrow{h_t} = LSTM_f(h_t, \overrightarrow{h_{t-1}}) \end{aligned}$$

4 $$\begin{aligned} & \overleftarrow{h_t} = LSTM_b(h_t, \overleftarrow{h_{t+1}}) \end{aligned}$$

The final hidden state representation at each time step is the concatenation of both directions:

5 $$\begin{aligned} h_t^{LSTM} = [\overrightarrow{h_t}; \overleftarrow{h_t}] \end{aligned}$$

where $h_t^{LSTM} \in {\mathbb {R}}^{2d}$, capturing both past and future dependencies.

#### Pooling and classification

To obtain a fixed-length representation for sentiment classification, we apply max pooling over the LSTM hidden states:

6 $$\begin{aligned} h_{pool} = \max _t h_t^{LSTM} \end{aligned}$$

Then, we concatenate this pooled output with the [CLS] embedding from BERT:

7 $$\begin{aligned} H_{final} = [h_{CLS}; h_{pool}] \end{aligned}$$

Finally, the classification layer maps this representation to sentiment classes using a fully connected layer with softmax activation:

8 $$\begin{aligned} {\hat{y}} = \text {Softmax}(W H_{final} + b) \end{aligned}$$

where $W$ and $b$ are trainable parameters, and ${\hat{y}}$ represents the predicted sentiment probabilities (e.g., Positive, Neutral, Negative).

This mathematical formulation highlights how BERT and LSTM work together to enhance the sentiment analysis of COVID-19 tweets, ensuring both deep contextual representation and sequential information retention.

#### Benefits of BERT-LSTM

BERT-LSTM effectively combines the strengths of BERT’s contextual word representations with LSTM’s ability to model long-term dependencies in sequential data. While BERT captures rich contextual meanings, it lacks explicit sequence modeling, which LSTM addresses by maintaining memory across the sequence, thereby improving sequential dependency modeling. This hybrid approach is particularly beneficial for sentiment analysis of tweets, as social media text is often informal and context-dependent, requiring both deep feature extraction and sequential understanding. Moreover, BERT-LSTM outperforms traditional ML models such as DTC, MNB and RFC, as well as standalone DL models such as BERT, by leveraging both powerful feature representations and sequential learning, ultimately leading to higher classification accuracy.

### Fine-tuning process

In order to maximize the performance of the suggested model BERT-LSTM and other deep learning models for the particular task at hand, we fine-tune them in our trials. In order to adapt pre-trained models, like BERT, to a new task, fine-tuning is an essential step that involves further training the model on the target dataset at a lower learning rate. This procedure preserves the generic information gained from extensive pretraining while enabling the model to learn domain-specific features and modify its weights. In order to provide rich, context-aware embeddings for the input text, we employ the pre-trained BERT model (bert-base-uncased) as the encoder for the BERT-LSTM model. A bidirectional LSTM layer is then applied to these embeddings in order to identify any sequential dependencies in the data. After the LSTM reduces the dimensionality of the sequence output using a MaxPooling layer, the features are concatenated with the [CLS] token output from BERT and then go through a fully connected layer for classification. The AdamW optimizer is the first optimizer we create to fine-tune the BERT-LSTM model. Its learning rate is $5 \times 10^{-5}$ and its weight decay is $1 \times 10^{-4}.$ This ensures effective training with an emphasis on minimizing overfitting. When training for classification problems, the loss is calculated using the Cross-Entropy Loss function. The model’s parameters are updated via backpropagation over the course of 20 training epochs. After each epoch, the model’s generalization capacity is evaluated on a validation dataset as part of the fine-tuning process. To keep an eye on the model’s development, we measure important performance indicators including recall, accuracy, and F1 score. The best-performing model for our assessment is the one that produces the highest validation accuracy. In summary, fine-tuning the BERT-LSTM and BERT models involves adjusting the pre-trained weights to suit the specific characteristics of our target dataset, ultimately improving their performance for our text classification task.

### Hyperparameters

Tuning hyperparameters is essential for maximizing model performance. Based on previous research and practical assessment, we carefully chose and adjusted the hyperparameters for both the ML and DL models in this work. We trained RFC with a learning rate of 0.001 and DTC, MNB, and LRC ML models with a learning rate of 0.1. The SGD optimizer was used to train each ML model for 100 epochs. To guarantee effective training, a batch size of 32 was chosen. We used a BERT-LSTM architecture for the DL model, combining an LSTM layer for sequential learning with BERT for contextual feature extraction. With a weight decay of $1 \times 10^{-4}$, the AdamW optimizer was used to optimize the learning rate, which was set at $5 \times 10^{-5}$. For regularization, a 0.25 dropout rate was used. ReLU was employed as the activation function for the 768 hidden units in the LSTM layer. The sequence length was set to 128 for consistent text processing, and a batch size of 64 was selected for effective gradient modifications. In order to balance convergence and computational efficiency, the model was trained across 20 epochs. The model was then stored for assessment, and the optimal epoch was chosen. These hyperparameter choices were carefully evaluated to improve generalization across binary, three-class, and five-class sentiment classification tasks.

#### Explanation of hyperparameters

- **Learning rate**: The learning rate controls how much the model updates its parameters during training. For machine learning models such as Decision Tree Classifier (DTC), Multinomial Naïve Bayes (MNB), and Logistic Regression Classifier (LRC), a learning rate of 0.1 is used. In contrast, Random Forest Classifier (RFC) utilizes a learning rate of 0.001. For deep learning models, a much smaller learning rate of $5 \times 10^{-5}$ is chosen to maintain stability during fine-tuning.
- **Batch size**: The batch size defines how many samples are processed in one training iteration. Traditional ML models commonly use a batch size of 32. However, deep learning architectures like BERT-LSTM require a larger batch size of 64 to efficiently manage memory constraints during training.
- **Epochs**: This parameter indicates how many times the entire dataset is used for training. Machine learning models typically undergo 100 epochs to ensure thorough learning, whereas deep learning models like BERT-LSTM are trained for 20 epochs. The lower epoch count in deep learning is due to the high computational demands, and the best-performing epoch is selected based on validation results.
- **Optimizer**: The optimizer fine-tunes model parameters to minimize loss. Machine learning models generally rely on Stochastic Gradient Descent (SGD) for optimization. In contrast, deep learning models employ the AdamW optimizer, which dynamically adjusts the learning rate and is particularly suited for handling large-scale architectures such as BERT.
- **Weight decay**: This regularization technique helps prevent overfitting by penalizing large parameter values. It is especially important for deep learning models like BERT-LSTM, where a weight decay value of $1 \times 10^{-4}$ is applied.
- **Dropout rate**: Dropout is a method used to improve generalization by randomly deactivating a portion of neurons during training. In the BERT-LSTM model, a dropout rate of 0.25 is employed to mitigate overfitting.
- **Hidden units**: In deep learning, the number of hidden units determines the size of the hidden layers. The LSTM layer in the BERT-LSTM model contains 768 hidden units, aligning with the pre-trained hidden state size of BERT.
- **Activation function**: The activation function determines how neuron outputs are transformed. In the BERT-LSTM model, the ReLU (Rectified Linear Unit) function is applied, introducing non-linearity into the network.
- **Sequence length**: This parameter specifies the number of tokens processed in each input sequence. For BERT-LSTM, a sequence length of 128 is chosen, which is a standard setting for fine-tuning BERT in text classification tasks.

These hyperparameters have been carefully tuned to enhance the performance of both machine learning and deep learning models. The selected values, particularly for deep learning models, are based on prior research and experimental validation, ensuring a balance between computational efficiency and model effectiveness.

## Results

We report the findings and evaluate the sentiment analysis performance of both ML and DL models in this part. Comparing each model’s efficacy and accuracy in predicting sentiment labels (positive, negative, and neutral) on the Kaggle sentiment tweet dataset is the main objective of the evaluation. In order to highlight distinct elements of the classification findings, the models’ performance is evaluated using a variety of assessment criteria.

### Evaluation metrics

When evaluating the effectiveness of categorization models, evaluation metrics are essential. They offer numerical metrics that facilitate model comparison and assess a model’s ability to differentiate between classes. These metrics are particularly crucial for sentiment analysis, since we need to assess the model’s ability to handle imbalanced data, sensitivity to particular classes, and other crucial features of prediction in addition to its overall accuracy (Table 7).

**Table 7:** Evaluation metrics and their respective equations.

Metric	Equation
Accuracy	$\frac{TP + TN}{TP + TN + FP + FN}$
Precision	$\frac{TP}{TP + FP}$
Sensitivity (Recall)	$\frac{TP}{TP + FN}$
Specificity	$\frac{TN}{TN + FP}$
F1 Score	$2 \times \frac{Precision \times Sensitivity}{Precision + Sensitivity}$
Kappa	$\frac{P_o - P_e}{1 - P_e}$ where $P_o$ is observed agreement, and $P_e$ is expected agreement
MCC (Matthews Correlation Coefficient)	$\frac{TP \times TN - FP \times FN}{\sqrt{(TP + FP)(TP + FN)(TN + FP)(TN + FN)}}$
MAE (Mean Absolute Error)	$\frac{1}{N} \sum _{i=1}^N (y_i - {\hat{y}}_i)$
MSE (Mean Squared Error)	$\frac{1}{N} \sum _{i=1}^N (y_i - {\hat{y}}_i)^2$
RMSE (Root Mean Squared Error)	$\sqrt{\frac{1}{N} \sum _{i=1}^N (y_i - {\hat{y}}_i)^2}$

#### Explanation of variables

- $TP$: True positives - The count of actual positive instances that are correctly classified as positive.
- $TN$: True negatives - The count of actual negative instances that are correctly classified as negative.
- $FP$: False positives - The count of actual negative instances that are incorrectly classified as positive.
- $FN$: False negatives - The count of actual positive instances that are incorrectly classified as negative.
- $P_o$: Observed agreement - The fraction of total instances where the model’s predictions match the actual values.
- $P_e$: Expected agreement - The estimated proportion of times the classifier is expected to predict correctly if making random guesses.
- $N$: Total number of instances in the dataset.
- $y_i$: The actual class label of the $i$-th instance.
- ${\hat{y}}_i$: The predicted class label of the $i$-th instance.

### Experiment environment

An environment with high-performance computers was used for the experiments, equipped with an NVIDIA Tesla P100-PCIE-16GB GPU, providing efficient parallel processing capabilities for deep learning tasks. The system was configured with 100GB of total disk memory, 57GB of available disk space, and 29GB of RAM, ensuring smooth data processing and model training. The GPU operated with a driver version of 560.35.03 and CUDA version 12.6, facilitating optimized execution of ML and DL frameworks. With a power consumption of 26 W out of a maximum 250 W and a temperature of $38\,^{\circ }$C, the GPU remained within stable operational limits throughout the experimentation process. This setup enabled effective training and evaluation of various sentiment analysis models while ensuring computational efficiency and scalability.

### Performance analysis

In our experiment, we performed sentiment analysis using various ML and DL models, including DTC, MNB, RFC, LRC, BERT, and BERT-LSTM. The models were evaluated using two data balancing techniques: WOS and ROS. Among these, Our hybrid (BERT-LSTM) model achieved the highest performance across multiple evaluation metrics, showcasing its effectiveness in sentiment classification. Table 8 presents a comparative performance analysis of various ML and DL models for binary sentiment classification under two different data balancing techniques: WOS and ROS. The results indicate that among traditional ML models, RFC and LRC achieve relatively high accuracy, with RFC reaching 82.68% (WOS) and 88.03% (ROS). However, DL-based models outperform traditional approaches, with BERT and BERT-LSTM achieving the highest performance scores. Notably, BERT-LSTM consistently surpasses other models, obtaining the highest accuracy of 89.81% (WOS) and 92.10% (ROS), along with superior F1-score, precision, and sensitivity. The results suggest that incorporating an RCNN structure with BERT enhances the model’s ability to capture contextual dependencies and improve classification performance. Furthermore, the lower MAE, MSE, and RMSE values for BERT-LSTM confirm its robustness in sentiment classification.

**Table 8:** Performance analysis of ML and DL models on binary class sentiment.

Data balancing	Model	Accuracy	Precision	Sensitivity	Specificity	F1score	Kappa	MCC	MAE	MSE	RMSE
WOS	DTC	76.05	76.08	76.05	74.65	76.06	49.23	49.23	23.95	23.95	48.94
	MNB	78.12	78.61	78.12	73.70	77.02	50.51	52.52	21.88	21.88	46.78
	RFC	82.68	82.56	82.68	80.46	82.43	62.36	62.67	17.32	17.32	41.62
	LRC	84.67	84.92	84.67	81.86	84.28	66.20	67.09	15.33	15.33	39.15
	BERT	86.93	86.98	86.93	84.91	86.73	71.57	71.97	13.07	13.07	36.15
	BERT-LSTM	**89.81**	**90.62**	**89.81**	**90.61**	**89.89**	**79.37**	**79.97**	**10.19**	**10.19**	**31.92**
ROS	DTC	83.33	83.56	83.33	83.30	83.30	66.64	66.88	16.67	16.67	40.82
	MNB	78.05	78.05	78.05	78.04	78.05	56.09	56.09	21.95	21.95	46.85
	RFC	88.03	88.12	88.03	88.01	88.02	76.04	76.14	11.97	11.97	34.60
	LRC	85.36	85.41	85.36	85.37	85.36	70.73	70.77	14.64	14.64	38.26
	BERT	86.36	86.88	86.36	86.31	86.30	72.69	73.22	13.64	13.64	36.94
	BERT-LSTM	**92.10**	**92.10**	**92.10**	**91.50**	**92.07**	**83.53**	**83.58**	**7.90**	**7.90**	**28.10**

Signficant values are in bold.

The performance analysis of ML and DL models on three-class sentiment classification (Table 9) shows that BERT-LSTM outperforms all other models in both WOS and ROS settings. Under WOS, BERT-LSTM achieves the highest Acc (88.52%), Prec (88.79%), Sen (88.52%), and Spec (93.72%), with an F1 (88.47%), Kappa (81.47%), and MCC (81.68%), significantly reducing MAE (13.95), MSE (18.90), and RMSE (43.48). BERT and LRC also show strong results, with BERT attaining 83.27% Acc and LRC reaching 80.20% Acc. Traditional models like DTC, MNB, RFC perform moderately, with RFC leading among them at 73.13% Acc. With ROS, BERT-LSTM further improves, achieving Acc (89.47%), Prec (89.80%), Sen (89.47%), Spec (94.10%), and F1 (89.44%), maintaining a strong Kappa (82.93%) and MCC (83.15%), while further reducing errors (MAE: 13.24, MSE: 18.67, RMSE: 43.21). RFC (82.84%) and BERT (83.36%) follow closely, while DTC (77.29%) and MNB (69.93%) see moderate performance. Overall, BERT-LSTM consistently delivers the best results, showcasing its robustness in sentiment classification. ROS improves model performance across the board, particularly for ML-based models like RFC and DTC.

**Table 9:** Performance analysis of ML and DL models on three class sentiment.

Data balancing	Model	Accuracy	Precision	Sensitivity	Specificity	F1score	Kappa	MCC	MAE	MSE	RMSE
WOS	DTC	63.36	63.84	63.36	80.95	63.53	42.49	42.53	55.24	92.42	96.14
	MNB	68.33	68.89	68.33	82.14	65.88	47.14	48.45	47.60	79.47	89.14
	RFC	73.13	72.99	73.13	85.74	73.02	57.15	57.19	40.23	66.95	81.82
	LRC	80.20	80.04	80.20	89.49	80.07	68.37	68.42	28.75	46.67	68.31
	BERT	83.27	83.32	83.27	91.25	83.28	73.51	73.53	24.98	41.47	64.39
	BERT-LSTM	**88.52**	**88.79**	**88.52**	**93.72**	**88.47**	**81.47**	**81.68**	**13.95**	**18.90**	**43.48**
ROS	DTC	77.29	77.07	77.29	88.65	76.85	65.94	66.22	35.57	61.30	78.29
	MNB	69.93	69.95	69.93	84.96	69.90	54.89	54.91	40.49	61.32	78.30
	RFC	82.84	82.92	82.84	91.42	82.59	74.26	74.53	26.01	43.70	66.11
	LRC	79.92	80.15	79.92	89.96	79.88	69.88	70.03	26.83	40.34	63.52
	BERT	83.36	83.84	83.36	91.69	83.38	75.05	75.26	23.22	36.37	60.31
	BERT-LSTM	**89.47**	**89.80**	**89.47**	**94.10**	**89.44**	**82.93**	**83.15**	**13.24**	**18.67**	**43.21**

Signficant values are in bold.

Table 10 provides the performance evaluation of ML and DL models for five-class sentiment classification and highlights the superiority of BERT-LSTM over other models across both WOS (Without Oversampling) and ROS (Random Oversampling) settings. Under WOS, BERT-LSTM achieves the highest accuracy (81.65%), precision (82.02%), sensitivity (81.65%), specificity (95.25%), and F1-score (81.71%), demonstrating its robustness. It also records the best Kappa (76.66%) and MCC (76.71%) scores while maintaining the lowest MAE (35.81), MSE (83.89), and RMSE (91.59). Traditional ML models such as RFC (53.7%) and LRC (58.03%) show moderate performance, while BERT (71.21%) significantly outperforms them. With ROS, overall performance improves, particularly for ML models like RFC (71.24%) and LRC (64.82%). However, BERT-LSTM maintains its lead, further increasing its accuracy to 81.78%, with precision (82.19%), sensitivity (81.78%), specificity (95.28%), and F1-score (81.85%). It also achieves slightly better Kappa (76.81%) and MCC (76.86%) while reducing error metrics (MAE: 35.31, MSE: 82.49, RMSE: 90.82). The ROS setting enhances model performance, demonstrating the benefits of oversampling in handling class imbalance. Overall, BERT-LSTM remains the most effective model, consistently outperforming both ML-based models and other transformer-based architectures in sentiment classification.

**Table 10:** Performance analysis of ML and DL models on five class sentiment.

Data balancing	Model	Accuracy	Precision	Sensitivity	Specificity	F1score	Kappa	MCC	MAE	MSE	RMSE
WOS	DTC	45.33	45.43	45.33	85.9	45.28	30.16	30.19	86.59	171.9	131.11
	MNB	47.11	52.4	47.11	85.91	46.07	30.44	31.62	74.13	124.38	111.52
	RFC	53.7	54.61	53.7	87.97	53.52	40.58	40.79	66.11	115.91	107.66
	LRC	58.03	58.77	58.03	89.09	58	46.16	46.3	56.26	89.88	94.81
	BERT	71.21	71.62	71.21	92.53	71.32	63.24	63.29	38.4	60.97	78.09
	BERT-LSTM	**81.65**	**82.02**	**81.65**	**95.25**	**81.71**	**76.66**	**76.71**	**35.81**	**83.89**	**91.59**
ROS	DTC	66.6	65.21	66.6	91.65	65.61	58.27	58.41	52.58	103.3	101.64
	MNB	53.91	52.29	53.91	88.48	52.6	42.41	42.62	69.94	134.55	116
	RFC	71.24	70.21	71.24	92.81	69.83	64.06	64.48	41.64	75.57	86.93
	LRC	64.82	63.69	64.82	91.21	63.71	56.04	56.29	48.18	81.84	90.46
	BERT	69.85	70.46	69.85	92.17	69.94	61.51	61.58	40.37	64.42	80.26
	BERT-LSTM	**81.78**	**82.19**	**81.78**	**95.28**	**81.85**	**76.81**	**76.86**	**35.31**	**82.49**	**90.82**

Signficant values are in bold.

#### Graphical analysis

**Fig. 2:**
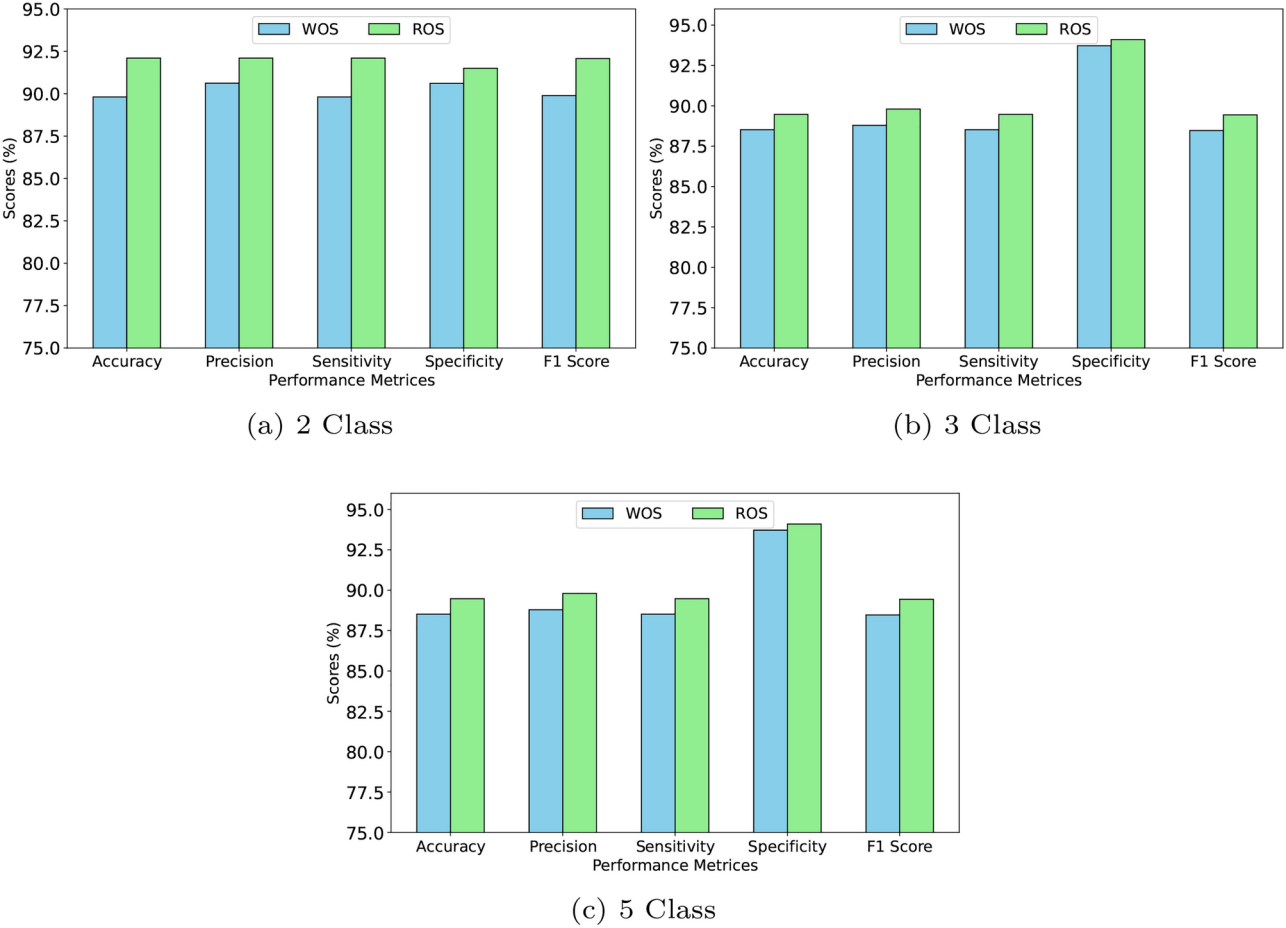
Performance analysis of BERT-LSTM model on sentiment dataset.

Figure 2 gives a graphical depiction of the performance of the BERT-LSTM model under WOS and ROS conditions for binary, three-class, and five-class sentiment classification. The depiction stresses the role of ROS in boosting accuracy, precision, sensitivity, specificity, and f1score. The results suggest that ROS boosts the model’s capacity to grasp complex patterns, resulting to enhanced classification accuracy across all emotion classes. The comparison further demonstrates that BERT-LSTM with ROS routinely beats its WOS equivalent.

#### Classification reports analysis

The classification reports for the BERT-LSTM model on sentiment analysis across binary, three-class, and five-class classifications demonstrate its effectiveness in capturing sentiment distinctions provided in Table 11. For the binary classification, the model achieves good accuracy, recall, and F1-score for both positive and negative feelings, with Negative sentiment (F1 = 93.43%) significantly outperforming Positive sentiment (F1 = 90.10%). In the three-class classification, the model maintains strong performance across all sentiment categories, with Neutral sentiment (F1 = 86.87%) showing slightly lower recall than Negative (F1 = 89.82%) and Positive (F1 = 90.08%), indicating some difficulty in distinguishing neutral expressions. For the five-class classification, performance remains robust, though slightly lower due to the increased complexity. Neutral sentiment (F1 = 86.14%) retains high precision, while Extremely Positive (F1 = 84.68%) and Extremely Negative (F1 = 83.32%) achieve balanced precision-recall scores. However, Negative (F1 = 78.89%) and Positive (F1 = 79.59%) show slightly reduced effectiveness, reflecting the challenges of fine-grained sentiment distinctions. These results indicate that the BERT-LSTM model performs exceptionally well across different sentiment classifications, with higher recall for strongly polarized sentiments and more difficulty in distinguishing neutral expressions, particularly in multi-class settings.

**Table 11:** Classification reports of BERT-LSTM model on sentiment analysis.

No. of Class	Class	Precision	Recall	F1score
2	Negative	92.18	94.72	93.43
	Positive	91.98	88.29	90.10
3	Neutral	92.67	81.74	86.87
	Positive	93.16	87.19	90.08
	Negative	85.54	94.55	89.82
5	Neutral	92.90	80.29	86.14
	Positive	77.18	82.15	79.59
	Extremely negative	79.66	87.33	83.32
	Negative	78.81	78.96	78.89
	Extremely positive	87.39	82.14	84.68

#### Confusion matrix analysis

The confusion matrix for the BERT-LSTM model on sentiment analysis gives a thorough perspective of its performance across multiple classes presented in Table 12. In the binary classification situation, the model exhibits good performance with 2133 properly categorized Negative cases and 1365 correctly classified Positive instances. The misclassification of 119 Negative samples as Positive and 181 Positive samples as Negative suggests some overlap in these categories. For the three-class categorization, the model showed strong capacity to discriminate between Neutral, Positive, and Negative attitudes. Neutral emotion is mostly confused with Positive and Negative samples, with 506 Neutral samples misclassified as Negative and 32 as Positive. Positive emotion is also well-classified, with just 18 misclassified as Neutral and 180 as Negative. Negative sentiment is largely properly detected, with 22 misclassifications as Neutral and 67 as Positive.

In the five-class classification, the confusion matrix becomes more granular, reflecting the increased complexity of sentiment categories. Neutral sentiment is often confused with Positive and Negative, with 497 Neutral samples misclassified as Positive and 61 as Negative. Positive sentiment shows some misclassification as Extremely Positive (63 samples) and Negative (85 samples). Extremely Negative sentiment is fairly well classified with 517 correct classifications but has some overlap with Negative (126 samples) and a few with Positive and Extremely Positive. Negative and Extremely Positive sentiments are mostly well-separated, with a few misclassifications occurring across neighboring classes, particularly with Negative being misclassified as Extremely Positive and vice versa. Overall, the confusion matrix highlights the model’s capacity to differentiate between major sentiment varieties while showing some confusion between adjacent sentiment labels, particularly when finer distinctions are needed.

**Table 12:** Confusion matrix of BERT-LSTM model on sentiment analysis.

No. of class	Class	Negative	Positive			
2	Negative	2133	119			
	Positive	181	1365			

		Neutral	Positive	Negative		
3	Neutral	506	32	81		
	Positive	18	1348	180		
	Negative	22	67	1544		

		Neutral	Positive	Extremely Negative	Negative	Extremely Positive
5	Neutral	497	57	2	61	2
	Positive	18	778	3	85	63
	Extremely negative	3	3	517	68	1
	Negative	16	72	126	822	5
	Extremely positive	1	98	1	7	492

## Discussion

The results of this study demonstrate that BERT-LSTM outperforms both traditional ML models (DTC, RFC) and other DL (BERT) models by achieving superior accuracy and robust metrics across balanced datasets. The incorporation of ROS significantly improved model performance, addressing class imbalance effectively. This highlights the potential of hybrid approaches, like BERT-LSTM, in handling nuanced sentiment classification tasks. These findings underscore the importance of advanced preprocessing and balanced training in achieving reliable sentiment analysis outcomes.

### Interpretation of the results

The findings of the proposed BERT-LSTM model illustrate its usefulness in the sentiment analysis of COVID-19 tweets, demonstrating greater accuracy compared to state-of-the-art (SOA) techniques. Table 13 shows a comparison study, demonstrating the advantages of the proposed approach over previous methodologies. The BERT-LSTM model, trained with ROS for data balancing and BERT embeddings for feature extraction, achieved 92.10% accuracy for binary classification and 89.47% accuracy for three-class sentiment classification. These results significantly surpass traditional machine learning models such as TF-IDF-based Stacking^15,17^, BLSTM^18^, and DL models like BERT^16^ and SARCOVID^19^. The higher performance of BERT-LSTM can be ascribed to its capacity to capture contextual semantics using BERT embeddings, which maintain the meaning of words dependent on their surrounding context. Additionally, the LSTM layer efficiently represents long-range relationships in textual input, enabling the model to handle complicated verbal patterns such as sarcasm, negations, and contextual changes. In contrast, prior SOA approaches depending on TF-IDF, Word2Vec, or Doc2Vec fail to completely capture the complex semantic patterns present in social media material. For instance, Bacsarslan et al.^14^ implemented a stacking ensemble model using Doc2Vec feature extraction and achieved 89.00% accuracy, which is comparable but slightly lower than our three-class classification result. Similarly, Jayapermana et al.^15^ employed TF-IDF-based Stacking, reaching 86.00% accuracy, while Bacsarslan et al.^17^ achieved 86.40% accuracy with a Word2Vec-based Stacking approach. Although stacking methods enhance classification performance, they do not fully leverage the contextual representation power of BERT, which contributes to the superior results of our model. Furthermore, Dobler et al.^16^ implemented a BERT-based model without LSTM and obtained 73.50% accuracy, indicating that integrating LSTM enhances sentiment classification by leveraging both contextual word representations and sequential dependencies. Similarly, Tareq et al.^18^ employed BLSTM, achieving 86.15% accuracy, which is still lower than our BERT-LSTM approach. Balaji et al.^19^ introduced SARCOVID, a model utilizing TF-IDF and ensemble techniques, but it achieved only 61.00% accuracy in sarcasm detection, demonstrating the limitations of traditional feature extraction methods in handling nuanced sentiment expressions in tweets.

Overall, the experimental findings validate the efficacy of the BERT-LSTM model in accurately classifying COVID-19-related sentiments, reinforcing the necessity of integrating context-aware embeddings and sequential learning mechanisms for improved sentiment analysis on social networks.

**Table 13:** Comparison analysis with state of art (SOA) works.

Author	Data balancing	Feature extraction	Model	No. of class	Accuracy (%)
Bacsarslan et al.^14^	–	Doc2Vec	Stacking	3	89.00
Jayapermana et al.^15^	–	TF-IDF	Stacking	3	86.00
Dobler et al.^16^	–	TF-IDF	BERT	3	73.50
Bacsarslan et al.^17^	–	Word2Vec	Stacking	3	86.40
Tareq et al.^18^	–	–	BLSTM	3	86.15
Balaji et al.^19^	–	TF-IDF	SARCOVID	2	61.00
Our study	ROS	BERT	BERT-LSTM	2	92.10
Our study	ROS	BERT	BERT-LSTM	3	89.47

### Implications of the study

The outcomes of this study have major significance for public sentiment analysis, crisis management, and decision-making processes in the context of COVID-19 and future global health emergencies. By leveraging BERT-LSTM, the proposed approach provides a more accurate and context-aware sentiment classification framework, which can be utilized by government agencies, healthcare organizations, and policymakers to assess public opinion, detect misinformation, and enhance crisis communication strategies. Unlike traditional ML models that rely on TF-IDF and shallow classifiers, the integration of BERT embeddings ensures a deeper understanding of linguistic nuances, enabling the detection of sarcasm, emotional shifts, and context-dependent sentiment variations. Moreover, the study addresses class imbalance through ROS, improving fairness and reliability in sentiment classification. These advancements make the model adaptable to broader applications beyond COVID-19, including political sentiment analysis, consumer behavior prediction, and mental health monitoring on social networks. Additionally, businesses and social media platforms can leverage this framework to refine customer feedback analysis and content moderation, fostering more responsive and data-driven decision-making. By demonstrating the effectiveness of DL in sentiment analysis, this study paves the way for further research into multi-modal sentiment analysis, cross-lingual adaptability, and real-time sentiment tracking, contributing to the evolution of natural language processing in social media analytics.

### Research questions validation

#### Q1 Validation

The effectiveness of preprocessing techniques is crucial for ensuring high-quality sentiment analysis of COVID-19-related tweets. In this study, a structured text preprocessing pipeline was implemented, including Unicode normalization to remove accents and special characters, contraction expansion to convert words and emoji conversion to preserve sentiment meaning. The text was standardized to lowercase, and social media artifacts such as URLs, mentions, and hashtags were removed. Tokenization split text into individual words, while stopword removal excluded common words but retained important negations. Lemmatization reduced words to their base forms for better generalization, and whitespace and special character cleanup ensured clean formatting. These preprocessing techniques improved the quality of the data, allowing the model to more effectively analyze and classify the sentiments expressed in COVID-19-related tweets, leading to higher accuracy and precision in sentiment prediction tasks.

#### Q2 validation

The BERT-LSTM model outperformed both traditional ML models and other DL models in the sentiment classification of COVID-19-related tweets. For binary classification, the BERT-LSTM model achieved an accuracy of 92.10%, which was notably higher than the performance of traditional models like DTC and RFC. In comparison to other deep learning models, including BERT, the hybrid BERT-LSTM model demonstrated superior performance in terms of accuracy, precision, sensitivity, and specificity. Specifically, the model achieved 89.47% accuracy for three-class classification and 81.78% for five-class classification, showcasing the effectiveness of combining BERT’s robust feature extraction with LSTM’s sequential learning capability for sentiment analysis of COVID-19-related tweets.

#### Q3 validation

The discoveries from this study have influential practical implications for public health communication and combating misinformation on social media. By accurately classifying sentiments surrounding COVID-19, the BERT-LSTM model can provide real-time insights into public perception. For example, negative sentiments identified by the model could highlight areas where misinformation or public fear may be widespread. On the other hand, tracking positive and neutral sentiments could provide insights into the effectiveness of public health messaging. These insights enable health authorities to refine their communication strategies and address emerging issues more proactively. Moreover, the ability to analyze large volumes of tweets efficiently allows for timely responses to misinformation, ensuring that health campaigns are better targeted and more effective in influencing public opinion. This demonstrates the value of automated sentiment analysis tools in guiding public health strategies and addressing misinformation on social media platforms.

### Limitations

One aspect not explored in this framework is the inclusion of data from a wider range of geographical regions. Additionally, while the current methodology focuses on sentiment analysis using standard features, exploring more advanced linguistic constructs such as sarcasm or irony could further improve the model’s accuracy in understanding complex social media expressions. Furthermore, the framework does not yet incorporate real-time, which could be beneficial in tracking sentiment trends as they evolve over time.

### Future work

In future work, the sentiment analysis framework can be expanded to support multilingual sentiment analysis, enabling the classification of tweets in different languages. Additionally, incorporating temporal features such as tweet timestamps could allow for tracking sentiment trends over time. Moreover, the generalization of the model to other domains, such as political discussions or other public health issues. The framework may be made more adaptable and durable for a greater variety of applications by optimizing the model on domain-specific data.

## Conclusion

This study explores sentiment analysis of COVID-19-related discussions on social networks using a BERT-LSTM model, demonstrating its effectiveness in capturing contextual and nuanced sentiments. The proposed framework outperforms existing state-of-the-art methods by leveraging BERT embeddings for rich feature extraction and LSTM for sequential learning, achieving high accuracy in binary and multi-class sentiment classification. The integration of ROS addresses class imbalance, ensuring more reliable sentiment predictions. Comparative analysis with previous studies highlights the model’s superior performance in handling complex linguistic patterns, sarcasm, and sentiment shifts in online discourse. The findings emphasize the significance of DL-based sentiment analysis in understanding public perception, aiding policymakers, healthcare organizations, and researchers in misinformation detection, crisis management, and social media monitoring. Moreover, the study lays the foundation for future enhancements, such as multilingual sentiment analysis, real-time sentiment tracking, and hybrid DL architectures, to further improve adaptability and accuracy. By advancing natural language processing and DL techniques, this study donates to the growing field of social media analytics, offering an effective and powerful solution for analyzing evolving online conversations.

## Data Availability

The selected datasets are sourced from free and open-access sources such as The COVID-19 Tweets dataset https://www.kaggle.com/datasets/datatattle/covid-19-nlp-text-classification.
